# Applying the science of learning to EdTech evidence evaluations using the EdTech Evidence Evaluation Routine (EVER)

**DOI:** 10.1038/s41539-023-00186-7

**Published:** 2023-09-06

**Authors:** Natalia Kucirkova, Garvin Brod, Nadine Gaab

**Affiliations:** 1 University of Stavanger, Learning Environment Centre, Stavanger, Norway; 2 The Open University, Milton Keynes, UK; 3 DIPF | Leibniz Institute for Research and Information in Education & IDeA Center for Research on Individual Development and Adaptive Education of Children at Risk, Frankfurt, Germany; 4 Department of Psychology, Goethe University Frankfurt, Frankfurt, Germany; 5 Harvard Graduate School of Education, Harvard University, Cambridge, MA USA

**Keywords:** Education, Human behaviour, Policy

Following frustrations with the pandemic learning loss and inadequate online teaching, the EdTech (educational technology) industry has taken the central stage of educational evidence discussions. EdTech is an umbrella term to encompass apps, learning platforms and online courses designed with the explicit purpose to educate and advance learning. The availability and variety of these tools expanded significantly after the COVID19 school closures but only 16% of 1058 educators surveyed by EdWeek (2023) described EdTech as very effective in accelerating learning. Indeed, converging evidence shows that although EdTech has the potential to provide highly individualized and advanced learning options, it is not meeting its potential (yet) to positively impact children’s learning^1–3^.

Mental health and learning outcomes are closely related and both are affected by students’ use of EdTech^4^. The U.S. Food and Drug Administration and similar agencies in other countries review and approve therapies offered on the market, including game-based digital therapeutic devices. However, despite repeated calls, there is no equivalent certification and approval agency for EdTech^5^. There are various and complex reasons for this, including the rapid development and often uncritical adoption of technologies that outpace the global research capacity for rigorous testing of the effects of these technologies; the misalignment of incentive mechanisms for EdTech developers and researchers to collaborate on product development research; the lack of scientifically trained EdTech entrepreneurs and dedicated EdTech training for scientists and the lack of international, EdTech-specific evaluation standards.

Disciplinary differences in how the quality of an EdTech product is evaluated, further complicate the assessment efforts. For example, in psychology, the focus on measuring learning outcomes and assessing instructional features through media comparison research studies is pertinent for gauging EdTech’s impact on academic performance^6^. We are an interdisciplinary research team and aim to advance the field with an initial, easy-to-apply guidance for evaluating EdTech’s evidence claims based on scientific standards. Based on general principles of the science of learning in terms of methodological plurality and quality assurance criteria, we outline a simple evaluation routine to facilitate discussions of EdTech evidence among diverse stakeholders.

Evidence-based EdTech has been called for but is in short supply, as shown in recent government and industry reports. Out of a hundred most popular EdTech in US schools, only a quarter had evidence of research and positive impact^7^. Despite being very popular and widely used by children, EdTech products often lack research-based insights on how we learn, which has negative consequences for early education^8^. For example, Meyer et al. (2021) analyzed the 124 most-downloaded EdTech mobile apps and reported that most of them were judged to stimulate repetitive, distracting, and meaningless experiences with minimal learning value^9^.

There are several reasons why a majority of EdTech ventures do not rely on evidence-based, scientifically rigorous research to evaluate and drive their impact. One is that EdTech ventures, by virtue of being part of a competitive marketplace, are driven by Key Performance Indicators, level of funds raised, retention, profit margins, or product scalability. When sales take over evidence, learning outcomes are not reached. This problem leads to products being deployed in learning environments that may or may not be effective and may even have negative effects. Indeed, the negative effects, such as lower or no learning after the introduction of EdTech into public classrooms, were noted by recent governmental reports assessing the state-of-art in EdTech after the pandemic (e.g. Department of Education Report in the UK, 2022; GrunnDig report in Norway, 2023.)^10,11^

Furthermore, there is the issue of EdTech companies using data for monetization and commercialization purposes. Many EdTech advertised to children use data with persuasive design intended to motivate children to use the app for as long as possible and engage them in repetitive use without advancing their learning^12^. Furthermore, popular EdTech advertised to young children contain manipulative design features such as pressures for children to complete a game within a short time, difficulty to navigate the screen or artificially prolong children’s app use^13^.

A related issue impeding a system-wide orientation towards evidence is a disconnect in the EdTech funding and development. While the investor and funding community typically value impact metrics that are guided by scientific research principles, they do not have a unified approach to guide these efforts. Some use national standards of evidence available in individual countries (e.g. ESSA Standards of Evidence in the USA or Australian Standards of Evidence in Australia), while others have their own internal assessment criteria that they apply as part of due diligence process. Others employ commercial consultants to gauge the scientific basis of companies seeking investment with their own, often non-transparent, assessments.

The scientific consensus is that EdTech *can* have a strong positive impact on educational outcomes if there are certain conditions in place, including that the technologies are designed with learning principles in mind. Evidence for this proposition has been provided in meta-analyses of apps for early learning or digital reading apps^14,15^. One of the key reasons that commercial EdTech have a low evidence base is that they are often not developed by, or with, researchers. The misalignment between latest scientific evidence and EdTech design is a methodological one and a practical one^16^.

Practically, the advancement of ethical, evidence-based EdTech is a complex task that requires collaboration between EdTech funders, producers, scientists as well as users (teachers and children/adolescents in classrooms). EdTech products should provide a full disclosure on the stage of development/level of maturity in their design, development, implementation, and evaluation process for the respective product. In the evaluation process, schools, procurement teams and funders need to know how to assess EdTech’s evidence base. What criteria for the quality of provided evidence should be used in the assessment (e.g., methodological quality)? What questions should be asked in determining how EdTech developers view and apply evidence in their work (i.e., assessing the partners’ willingness to engage with research and scientists and their commitment to improving/learning as they develop their product)?

These questions do not have straightforward answers, but they can be systematically reflected upon with some guiding frameworks. There are many analysis questions to consider when making a conclusion about “what works” in education - even the largest educational clearinghouses (such as the What Works Clearinghouse (https://ies.ed.gov/ncee/wwc)) apply different evaluative standards and draw divergent recommendations about which educational programme is evidence-based^17^. This can be confusing for EdTech and should be routinely addressed with an evaluation approach, spanning foundational research, practice-informed basic research, and user-oriented research with direct applicability to policy and practice.

In developing such an evaluation routine, it is important to embrace methodological plurality that recognises the value of all types of research, without positioning RCT evidence as the best evidence for all EdTech. The principles of science of learning also emphasize a match between the method and the question—different designs and methods answer different research questions and there is no universally applicable hierarchy of research methods. Finally, it is important to adopt an evaluation routine that would not only evaluate an existing product but also advance a culture of evidence and learning at all stages of design—from developing the theory of change, to early testing and validation of their model, to promising models codifying their approach, to proven approaches poised for replication.

We propose The EdTech Evidence Evaluation Routine (EVER) as a simple guide to be applied in the evaluation of the evidence base of existing EdTech solutions and to guide the EdTech companies in growing their products’ evidence base. Table 1 outlines the evidence base and the evaluation approaches employed to test an EdTech product (rows) and the quality of their implementation (columns).

**Table 1:** The EdTech Evidence Evaluation Routine.

Quality assurance aspects/Evaluation approaches	Methodological quality	Outcome strength/predictive value	Generalizability	Ethics and transparency
Conceptual	rating	rating	rating	rating
Qualitative	rating	rating	rating	rating
Quantitative	rating	rating	rating	rating
Validation	rating	rating	rating	rating

EVER can be applied to the development of EdTech solutions, the evaluation of existing or planned products, and the investment in products. Thereby, products with poor or no evidence can be filtered out and conversely, more quality products will enter and/or remain in the EdTech market. Our intention is to encourage this cycle with EdTech created for assessment, intervention or edutainment (i.e. education coupled with entertainment) in K-12 education.

Indeed, EVER can be used for EdTech of any type, including those that are designed to promote foundational skills in literacy and math, those that aim to change learners’ behaviour, as well as those that combine assessment and intervention. EVER can be used at various stages of an EdTech’s lifecycle, including the pre-company stage as part of an accelerator or when mature companies look for additional funding. The strength of each of the criteria should be rated on a 0–5 point-scale for each of the cells, including the cells where the company has no activity.

*Methodological quality* denotes whether the evaluation methods used are appropriately executed, described and justified, and what the results show. It helps to answer questions such as “Is the rationale sound or logically flawed?”, “Can the chosen methodology speak to whether the EdTech works as intended?” and “Has the EdTech been tested in a sufficiently large target population?” *Outcome strength* denotes whether the EdTech has a sizable impact or predictive value. Impact is usually quantified as a significance measure or an effect size, which is a quantitative measure of the magnitude of the effect on a particular external measure. It helps to answer questions such as “How much of an effect does the EdTech have?” and “How accurate is the tool?” *Predictive value* can be quantified by sensitivity/specificity predictive validity and classification accuracy, which are quantitative measures of how good a tool is at correctly distinguishing groups/categories (e.g., with/without reading difficulties).

*Generalizability* can be defined as the extension of research findings and conclusions from a research study conducted on one selected sample population to the population (or a target population) at large. While a larger sample typically comes with a higher generalizability, it still needs to match the target population in terms of demographic characteristics, socio-cultural values, skills and abilities (i.e., it needs to be representative of the target population). It helps to answer questions such as “Can I be sure that the tool works for my students?” and “Will the tool be well-received in my market?” or “Who will the product be helpful for?”.

Finally, *Ethics and Transparency* ensure that the questions asked or the design of the EdTech and its purpose are ethical, as well as ensuring users’ well-being as well as broader contributions to social justice. It includes culturally-responsive approaches and a transparent use of participants’ data. It helps to answer questions such as: “Do users know which personal data are collected, used, or otherwise processed?”, “What are the data protection standards?”, and “Are users treated respectfully and is their dignity preserved?” There are different criteria for assigning scores in each of the quality assurance aspects with different types of evaluation methods. For example, the criteria to assess methodological quality of conceptual studies can be different from generalisability criteria in quantitative or qualitative studies.

The proposed EdTech Evaluation Routine can be used as a prompt for reflection when evaluating the evidence portfolio of diverse EdTech products, processes and initiatives. The synergistic model proposed through the evaluation process takes into account the benefits and limitations of different methodological approaches and can be applied in conjunction with local quality assurance assessments of EdTech (for example those applied at district or school level) as well as by EdTech developers in iterative product development. EVER is best used as part of formative evaluations; it is not intended to determine “good” or “bad” solutions but rather to offer a constructive template for addressing the current lack of EdTech evidence in the ecosystem.

The advent of generative AI, and the current lack of accountability measures that ensure the implementation of evidence-based criteria in children’s EdTech, mobilised international governments into action. Organisations offering rapid evaluations and research consultancy services for EdTech have emerged alongside increased academia-industry partnerships. The evaluation routine can be seen as a first step toward an international, open-access benchmark of EdTech evidence in various partnership models between researchers and the EdTech community. EVER can be used alongside internal company or non-profit research and national evaluation standards and should be supplemented with other frameworks that target cost-effectiveness, data privacy and teachers’ usability evaluations.

In conclusion, the Science of Learning is an interdisciplinary field of study with many diverse methodologies. The open-ended nature of EVER is intentional in that we wish to promote an equitable approach to EdTech evidence that acknowledges the limited access some, notably smaller start-ups from low and middle-income countries, have to research teams and testing possibilities in schools. We hope that the guidance within our preliminary EdTech Evaluation Routine can be used as a prompt for discussions about EdTech evidence across various stakeholder groups and be part of the mind shift necessary for promoting greater integration of science into EdTech design and thereby, better learning outcomes for our students.
